# The Chlorophyll a Fluorescence Modulated by All-Trans-β-Carotene in the Process of Photosystem II

**DOI:** 10.3390/ijms17060978

**Published:** 2016-06-21

**Authors:** Tianyu Li, Ye Zhang, Nan Gong, Zuowei Li, Chenglin Sun, Zhiwei Men

**Affiliations:** 1 Coherent Light and Atomic and Molecular Spectroscopy Laboratory, College of Physics, Jilin University, Changchun 130012, China; tianyu14@mails.jlu.edu.cn (T.L.); gongnan15@mails.jlu.edu.cn (N.G.); zuowei_li@163.com (Z.L.); chenglin@jlu.edu.cn (C.S.); 2 College of Plant Science, Jilin University, Changchun 130062, China; 13258880526@163.com

**Keywords:** chlorophyll a, fluorescence, all-trans-β-carotene, electron transfer

## Abstract

Modulating the chlorophyll a (Chl-a) fluorescence by all-trans-β-Carotene (β-Car) in the polarity and non-polarity solutions was investigated. The fluorescence intensity of Chl-a decreased as the concentration of β-Car increased. The excited electronic levels of Chl-a and β-Car became much closer owing to the solvent effect, which led to the electron transfer between both two molecules. A electron-separated pair Chl^−^·Chl^+^ that is not luminous was formed due to electron transfer. The solution of Chl-a and β-car in C_3_H_6_O was similar to the internal environment of chloroplast. We conclude that the polar solvent is good for the fluorescent modulation in photosystem II.

## 1. Introduction

Photosynthesis is the biological basis of all material and energy metabolism, and it is very important for the growth and development of plants. Fluorescence plays a much more important role in avoiding the chloroplasts absorption of light compared with photosynthesis digestion to reduce to a minimum loss of strong light. The chlorophyll a (Chl-a) fluorescence and photosynthetic rate were negatively correlated with each other under natural conditions [1,2]. Chlorophyll and carotenoids are both light-harvesting pigments, but chlorophyll plays an important role in the process of photosynthesis. Carotenoid is an alkene molecule that contains π-electron conjugated double bonds, and it has the function of light collection and protection, which is closely related to the chlorophyll molecules in the chloroplast structure [3]. The chemical structure of chlorophyll is such that it may gain or lose electrons easily, can absorb photons, and can transfer the excitation energy to the photosynthetic reaction center [4]. All-trans-β-Carotene (β-Car) plays a role in regulating the energy flow of Chl-a excited states in the photosynthetic antenna, being used in an energy dissipation route [5,6].

In this paper, we investigate the fluorescence characteristics of Chl-a and the function of β-Car on the quenching process of Chl-a fluorescence. The UV-visible absorption and fluorescence spectra for different concentrations of β-Car and Chl-a in organic solvents were measured. At a certain concentration range, the fluorescence quenching effect of Chl-a becomes better with the increase of the concentration of β-Car. The interactional mechanism between the π-electrons of β-Car and the Chl-a singlet state are discussed in terms of the process of quenching of Chl-a singlet electronic excited state.

## 2. Results and Discussion

**Figure 1:**
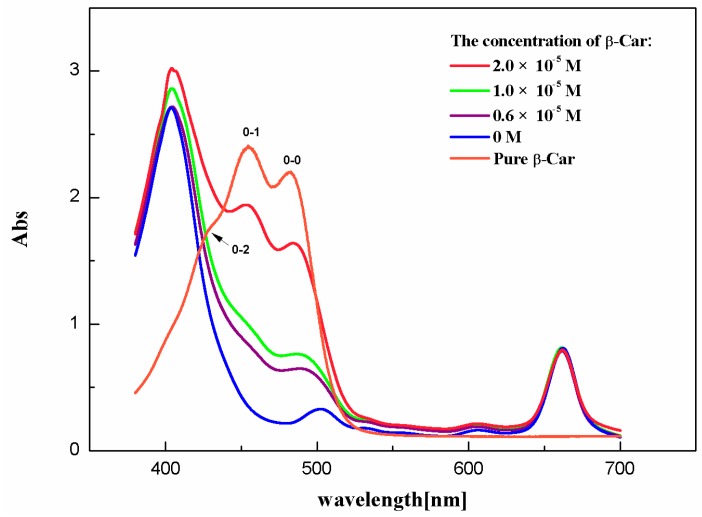
The absorption spectra of chlorophyll a (Chl-a and all-trans-β-Carotene β-Car in C_3_H_6_O solution.

Chl-a has two strong absorption bands: one is located within 640–660 nm in the red area, the other is located within 410–430 nm in the blue-violet light area. The absorption peaks of β-Car appear at 426, 453, and 485 nm. They are produced by allowing transitions of 1^1^Ag^−^(S_0_) → 1^1^Bu^+^(S_2_) [3,7]. Markings as shown in Figure 1 from left to right are 0-2, 0-1, and 0-0.

**Figure 2:**
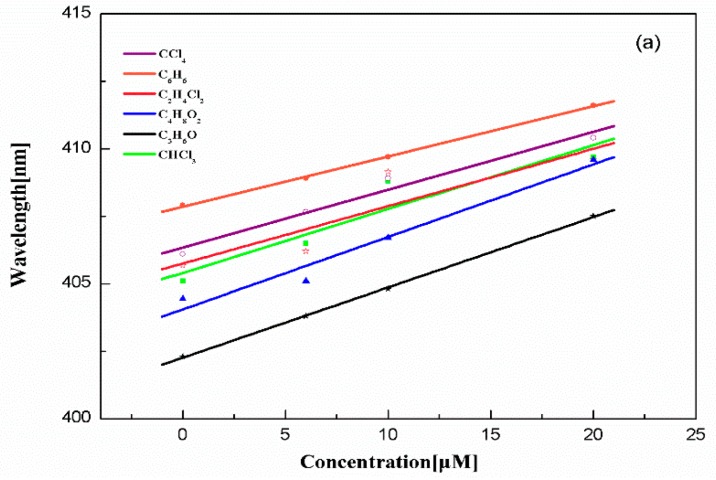

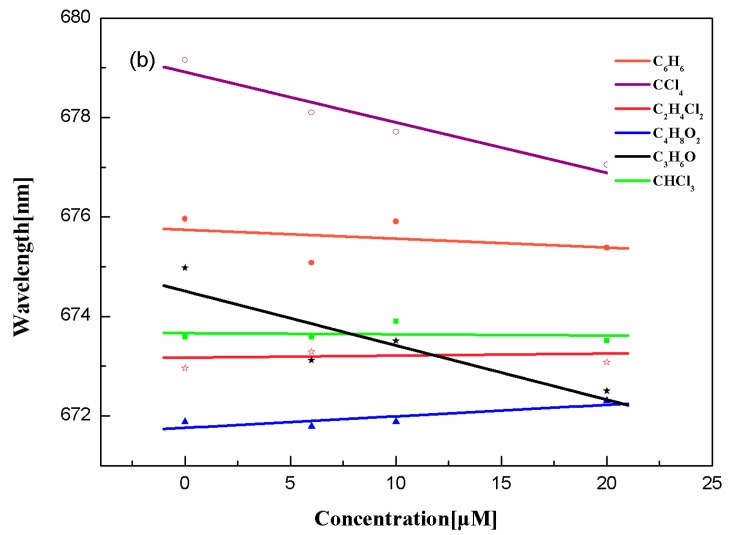
The dependence of Chl-a absorption (**a**) and fluorescence (**b**) wavelength on the concentration of β-Car in different solvents.

Figure 2 shows the absorption and the fluorescence positions of Chl-a change along with the concentration in polarity and non-polarity solvents. From Figure 2a, it shows that the absorption wavelength of Chl-a increases with the increased concentration of β-Car. Absorption band of Chl-a at 410 nm arises red-shifted because of the solvent effect, which results in the excited electronic levels of Chl-a and β-car becoming much closer, there is the lower electron energy level in the solute [8]. C_3_H_6_O appears to have the most obvious frequency shift. The wavelength of fluorescence decrease for C_3_H_6_O is illustrated in Figure 2b. Although the ground state and the excited state energy are both decreased, the effect of polar solvents on the excited states is stronger than that of the ground state. The energy difference between both levels becomes small [3,9]. Polar solvents should cause a red shift for π → π * transitions and a blue shift for n → π * transitions, especially for ketones [10,11]. The electron distribution in the molecule is asymmetric, and the intrinsic dipole moment is generated. Between the two molecules with intrinsic dipole moments, there will be intermolecular electrostatic interactions [12].

**Figure 3:**
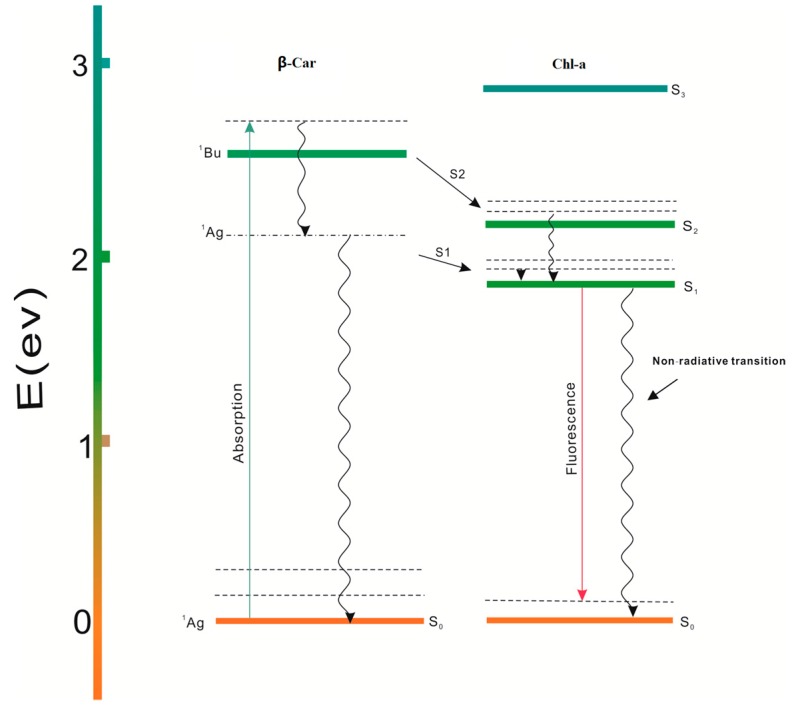
Scheme of excited electronic levels of Chl-a and β-Car.

Figure 3 shows that the levels is critical in energy transfer. It is believed that β-Car does not fluoresce [13] when excited in visible range. It is accepted that, instead of the strongly allowed ^1^Bu state, the lowest excited singlet electronic state of β-Car is a dipole forbidden ^1^Ag state at about 3450 cm^−1^ lower in energy [14]. The vibronic transition from the excited singlet state of ^1^Ag to the ground singlet state of β-car has an overlap with the absorption band of the Chl-a [15,16].

**Figure 4:**
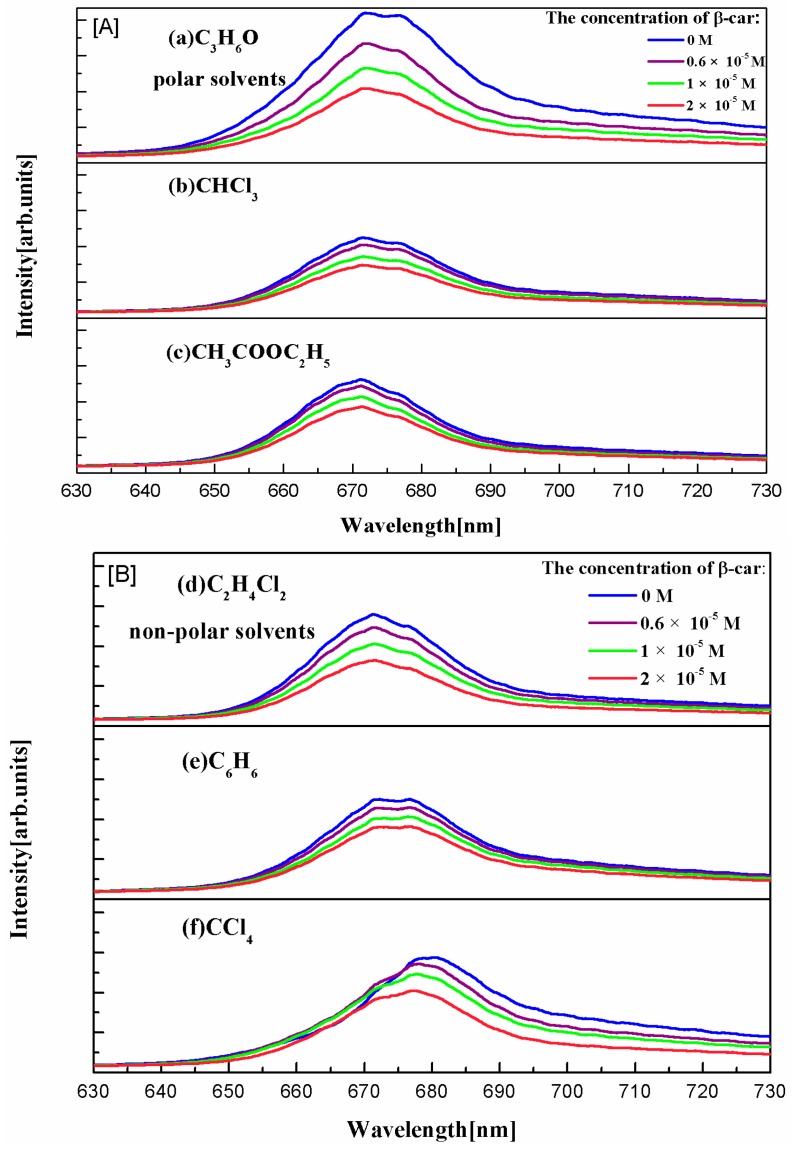
Fluorescence spectra of Chl-a in polar and non-polar β-Car solutions. ((**A**) are the polar solvets; (**B**) is the non-polar solvents. (**a**) C_3_H_6_O (**b**) CHCl_3_ (**c**) CH_3_COOC_2_H_5_ (**d**) C_2_H_4_Cl_2_ (**e**) C_6_H_6_ (**f**) CCl_4_).

Figure 4 shows the fluorescence spectra of Chl-a in polarity and non-polarity solvents. It is obvious that the fluorescence intensity of Chl-a decreases with the increase of the concentration of β-Car. On interaction of the excited singlet state of β-Car with Chl-a, the energy can be partitioned between energy transfer and an electron transfer process [17]. For the fact that the energy level of Chl-a is lower than that of β-Car, the electron transfer will dominate in the center of the reaction. Moreover, β-Car has a lower ionization potential. As a result, it is likely to become the electron donor, and Chl^−^ and Car^+^ generate during the whole process of electron transfer. The Chl^+^ is generated through a fast ground state reaction (charge transfer) between the Car^+^ and its nearby Chl-a. Therefore, the consequence of the process is a generation of the separated change pair Chl^+^·Chl^−^ [18,19]. The energy levels of the compound are close in the solution, so that the electron in the upper level will easily undergo a non-radiative transition to the lower level. Therefore, the fluorescence is associated with a red shift. The product of the experiment is non-emissive, which leads to the quenching of Chl-a fluorescence.

**Figure 5:**
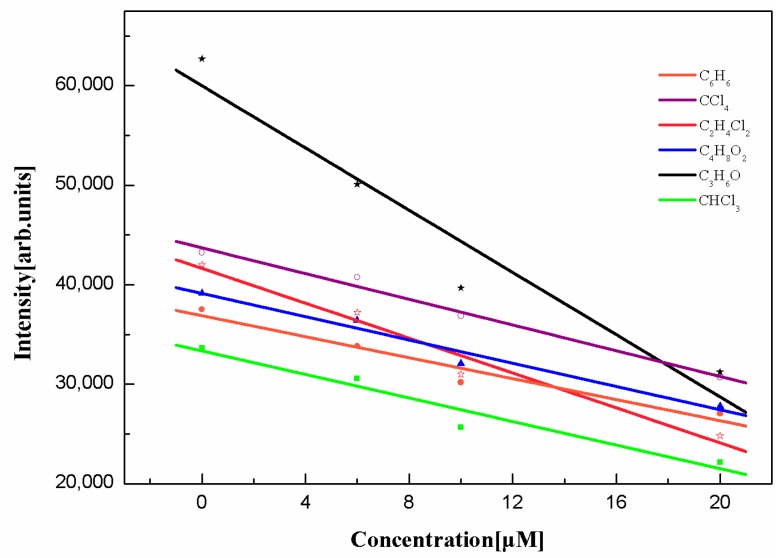
The dependence of fluorescence intensity on the concentration of β-Car in different solvents.

A more intuitive trend is shown in Figure 5, where the slope of C_3_H_6_O is the steepest. Polar solvent on the quenching effect is more obvious. The polarity of organic solvents used in the experiment from large to small is: C_3_H_6_O > CHCl_3_ > C_4_H_8_O_2_ > C_2_H_4_Cl_2_ > C_6_H_6_ > CCl_4_. Therefore, C_3_H_6_O is a relatively effective quenching agent in these organic solvents because the effect of polar solvents on the excited states is stronger than that of the ground state [20]. Quenching of fluorescence by β-Car protects the plants from strong light radiation. In a certain concentration range, the Chl-a fluorescence intensity decreases with the increase in the concentration of carotene, which improves the efficiency of photosynthesis. In lower plants, transfer from β-carotene to chlorophyll occurs in Photosystem I exclusively. In higher plants energy transfer from β-carotene to chlorophyll occurs in both Photosystem I and II. Light absorbed by β-carotene is transferred to chlorophyll with nearly 100% efficiency [21]. In addition, there is a huge quantity of inorganic salt and polar lipids in the chloroplast, and the polar environment is similar to the C_3_H_6_O solution that we used in the experiment [22,23].

## 3. Experimental Section

All samples contained the same Chl-a concentration of 6.7 × 10^−5^ M. The concentrations of β-Car are 0.6 × l0^−5^, 1 × l0^−5^, and 2 × 10^−5^ M, respectively. The nonpolar solvents used include C_6_H_6_, CCl_4_, and C_2_H_4_Cl_2_, and the polar solvents used include C_3_H_6_O CHCl_3_, and CH_3_COOC_2_H_5_. The exciting laser power was fixed at 30 mW with wavelengths of 532 nm. The quartz cell size is 100 × 10 × 45 mm. The fluorescent signal is collected perpendicular to the incident laser beam and is recorded by Ocean Optics (Maya 2000, Ocean optical Corporation, Dunedin, FL, USA). Absorption spectra were recorded on a double-beam UV-Vis spectrophotometer (TU-1901, Persee Co., Ltd., Beijing, China) with a step of 0.5 nm at room temperature.

## 4. Conclusions

In conclusion, β-Car can modulate the fluorescence of Chl-a in the solutions. The excited electronic levels of Chl-a and β-car became much closer owing to the solvent effect, which led to the electron transfer producing an electron-separated pair Chl^−^·Chl^+^ that is not luminous. The effect of polar solvents on the excited states is stronger than that of the ground state in C_3_H_6_O solution, which results in a better quenching effect, and the polar environment is very similar to the internal environment of chloroplast. The results provide references for optical protection of plants during the photosynthesis process.
